# Exploration of physical activity, sedentary behavior and insulin level among short sleepers

**DOI:** 10.3389/fendo.2024.1371682

**Published:** 2024-10-14

**Authors:** Yuquan Chen, Yanwei You, Mengxian Wei, Ping Yang, Qi Zhang, Xingzhong Li, Qun Zuo, Qiang Cao

**Affiliations:** ^1^ School of Public Health and Preventive Medicine, Faculty of Medicine, Nursing & Health Sciences, Monash University, Melbourne, VIC, Australia; ^2^ Division of Sports Science and Physical Education, Tsinghua University, Beijing, China; ^3^ Department of Pathology, Johns Hopkins University School of Medicine, Baltimore, MD, United States; ^4^ Department of Undergraduate, Taishan University, Taian, China; ^5^ Orthopedics Department, PLA Rocket Force Characteristic Medical Center, Beijing, China; ^6^ College of Public Health, Hebei University/Hebei Key Laboratory of Public Health Safety, Baoding, Hebei, China; ^7^ School of Pharmacy, Macau University of Science and Technology, Macao, Macao SAR, China; ^8^ Department of Earth Science, Kunming University of Science and Technology, Kunming, China

**Keywords:** physical activity, sedentary behavior, serum insulin, NHANES, short sleepers

## Abstract

**Background:**

Sufficient physical activity and sleep duration are essential for overall health. While one-third of the US population reports short sleep (<7 h), which is proven to link with negative health status. Current evidence on the relationship between physical activity, sedentary behavior, and serum insulin level in short sleep groups is limited.

**Methods:**

The National Health and Nutrition Examination Survey (NHANES) was used to conduct this cross-sectional study of 8,494 adults (NHANES) 2007–2018. Serum insulin was quantitatively tested by human insulin immunoassay. Short sleep conditions were defined as ≤7 h per night. Physical activity conditions, including work activity, recreational activity, and sedentary behavior, were self-reported in NHANES by the Physical Activity Questionnaire using a 7-day recall method. The main analyses utilized weighted linear regression models due to the complex multistage sampling design of NHANES. Subgroup analysis and the influence of different lipid indices were explored in this study. In addition, a sensitivity analysis of participants without diabetes was conducted.

**Results:**

In fully adjusted models, increased levels of work and recreational activity significantly reduced insulin levels, with β values 95% CI = -0.002 (-0.003, 0.001) and β values 95% CI = -0.008 (-0.012, -0.003), respectively. However, sedentary behavior was positively associated with insulin levels, with a β value 95% CI =0.022 (0.009, 0.034). The sensitivity analysis further confirmed the benefits of recreational activity in controlling insulin levels. Through sex stratification analysis, it seemed that physical activity was more obviously impacted in the male than female groups.

**Conclusions:**

Overall, our analysis demonstrates that in short sleepers, an increased level of work and recreational activity is beneficial to control the insulin level, and more sedentary time is harmful. However, this association might be discrepant in different sexes and different levels of lipid indices.

## Introduction

Sleep deprivation has been associated with the development and management of a number of chronic diseases, including depression, cognitive decline, type 2 diabetes, and coronary heart disease, and metabolic disorders such as insulin fluctuation (1–4). Sleep deprivation is also associated high rates of morbidity and mortality. It is concerning that nearly one-third of the U.S. population reports sleeping less than seven hours per night on average (5). Advancing occupational and social demands contribute to the continuous decline in sleep duration, and the knowledge for improving quality of life is still lacking among short sleepers.

Insulin, the body’s primary hormone, regulates glucose metabolism and peripheral tissue glucose uptake and utilization. Insulin levels rise rapidly after a meal, peaking 30 to 45 minutes after the meal’s onset (bolus/prandial), and then fall back to basal levels within an hour or so. In healthy individuals, basal insulin is secreted continuously at a low rate (concentrations of 5-15 μU/mL) (6). Maintaining normal blood glucose concentrations and keeping them within a narrow range (63-135 mg/dL [3.5-7.5 mmol/L]) is beneficial for people without diabetes (7). Insulin resistance (IR), characterized by the body’s reduced ability to respond to insulin, is a hallmark of type 2 diabetes mellitus (T2DM), where insulin release may be significantly decreased or absent (8, 9). Another possible scenario in T2DM is that insulin release may be significantly decreased or completely absent (6). Fasting hyperglycemia occurs as a result of inadequate basal insulin secretion to maintain normal fasting plasma glucose concentrations or as a result of the appearance of insulin resistance (10–12).

While numerous studies have highlighted the health risks of sedentary behavior (SB) and physical inactivity (PI) (13), a significant portion of the American and global population continues to engage in high levels of SB/PI and low levels of physical activity (PA), often coupled with inadequate sleep (14–16). Especially, current studies showed that the benefits of PA existed in short-sleep population (17, 18). Notably, diabetes or prediabetes is closely associated with high SB/PI levels. Data from the National Health and Nutrition Examination Survey (NHANES) 2003 - 2006 indicated a negative association between physical activity and prediabetes prevalence in middle-aged U.S. adults, independent of adiposity (19). Another study also pointed out that sedentary lifestyles are important drivers of the current global diabetes epidemic (20). Owing to the high levels of SB/PI having impacts on basal insulin levels, both of them can increase the risk of diabetes significantly. Long-term sedentary behavior will lead to the body secreting a large amount of insulin, and its excessive use and consumption will further lead to basal insulin deficiency or IR. One study used NHANES to support this view and found that SB might be an important modifiable determinant of concentrations of insulin, and simultaneously, a significant positive correlation was found between SB and PI (21). Christian K Roberts also showed that PI was an important reason for metabolic syndrome and insulin resistance (9).

PA demands fuel mobilization and oxidation, with insulin’s effects on fuel storage being suppressed during exercise. This is achieved by reducing insulin release during PA and initiating systemic and local fuel mobilization (22). The effects of insulin on fuel storage are diminished during PA, which is primarily accomplished by blocking the release of insulin during PA and triggering both systemic and local fuel mobilizing processes (22). After the PA process, it is necessary to increase the insulin sensitivity of exercise muscles to increase glycogen storage. Hence, there is plentiful evidence that PA can prevent the abnormal increase in insulin levels and the occurrence of IR (22, 23). Several studies have also confirmed that PA could reduce the occurrence of abnormal increases in triglycerides (TG), high-density lipoprotein (HDL), low-density lipoprotein (LDL), and total serum cholesterol (TC). Those are all higher risks of developing diabetes, heart disease, or other illnesses (24–26). Regarding the specific movement mode, moderate-to-vigorous PA has proven particularly effective. For the short-sleep population, current studies have reported that PA can induce positive effects on hemodynamic changes and cognitive health (27, 28). Moderate-to-vigorous PA, in particular, has proven effective in enhancing these health outcomes.

The *2008 Physical Activity Guidelines for Americans* recommend engaging in at least 150 to 300 minutes of moderate-intensity aerobic activity each week (29). This level of activity has been shown to effectively improve ‘poor’ sleep quality (30–32). However, the relationship between PA, SB, and insulin levels in populations with short sleep duration has been less explored.

The present study aims to explore the relationship between physical activity, sedentary behavior, and insulin levels in individuals with short sleep durations. We hypothesize that increased daily physical activity and reduced sedentary behavior are associated with improved serum insulin levels in this population. This research seeks to provide insights into the clinical significance of these lifestyle factors for short sleepers, potentially informing interventions to improve their metabolic health (33).

## Methods

### Study population

The NHANES is a National Center for Health Statistics program that is part of the Centers for Disease Control and Prevention (CDC). A structured household interview evaluated participants, and a standardized physical examination in mobile examination centers (MEC) with room for blood draws and measurement. The NHANES was designed to represent the population of the United States, which has become a continuous program since 1999 using a stratified, multistage probability sampling design; the NHANES survey was conducted every two years. More information on the sampling procedure is available on the website (http://www.cdc.gov/nchs/nhanes/about_nhanes.htm#intro). The present study included participants aged 18 years and older in six NHANES cycles (2007-2008, 2009-2010, 2011-2012, 2013-2014, 2015-2016, 2017-2018). Sleep duration on a usual weekday or workday was self-reported by participants. Referring to previous literature (34, 35), a short sleep condition was defined as ≤7 h per night.

### Outcome: insulin level

In the NHANES 2011-2012 data collection cycles, the University of Minnesota conducted insulin testing using the Roche Elecsys 2010 immunoassay. Between 2013 and 2018, insulin testing was performed by the University of Missouri-Columbia, utilizing a two-site immunoenzymetric assay with the Tosoh AIA System Analyzer. Within the AIA-PACK, insulin was bound to a monoclonal antibody immobilized on a solid permanent magnet phase and an enzyme-labeled monoclonal antibody. The magnetic beads were then incubated with the fluorogenic substrate 4-methylumbelliferyl phosphate to remove any unbound protease mabs. The quantity of enzyme-labeled specific antibodies that bind to the beads is proportional to the insulin concentration of the sample. The densities of unknown compounds were characterized using a constructed standard curve. More information about the quality control and test process can be found in a detailed description at http://www.cdc.gov/nchs/nhanes/.

### Exposure: PA status and SB

The independent variables of this study were physical activity conditions, including work and recreational activity, and sedentary behavior. The information on physical activity was self-reported in NHANES by the Physical Activity Questionnaire using a 7-day recall method. According to the questionnaire (36) provided by NHANES, work activity was defined as doing work and chores, such as cleaning the yard and trimming the sprays, which can be regarded as labor activities. Referring to previous literature (37–39), recreational activity was referred to as leisure time activities, including sports and exercise. The participants reported the duration and frequency of each type of physical activity at vigorous and moderate levels of intensity during a typical week. Subsequently, we summed the total number of minutes of physical activity at each intensity level to determine the remaining minutes of work and recreation. Sedentary behavior was measured as sitting time, defined as daily time spent “sitting at work, at home, getting to and from places, or with friends, including time spent sitting at a desk, traveling in a car or bus, reading, playing cards, watching television, or using a computer” and was assessed with the question “How much time do you usually spend sitting on a typical day?”

### Covariates

Sociodemographic characteristics included age, sex race/ethnicity (non-Hispanic White, non-Hispanic Black, Mexican American, other race), marital status (never married, married/living with partner, widowed/divorced) and education (< high school, High school or equivalent, and > High school), family income-to-poverty ratio (<1.3, [1.3, 3.5), ≥3.5). BMI was calculated as weight (kg) divided by height squared (m^2^) and classified into 3 groups (<25, [25, 30), ≥30 kg/m^2^). Lifestyle factors included smoking status (never, former, and current), alcohol use (never, moderate, high). The physiologic screening tests fast glucose (FG), triglyceride (TG), total cholesterol (TC), high-density lipoprotein cholesterol (HDL-C), and low-density lipoprotein cholesterol (LDL-C) were measured in blood samples. Participants were instructed to fast for at least 8.5 hours prior to blood sample collection. TG, TC, HDL-C, and LDL-C levels in serum samples were measured in mg/dL. Additionally, blood pressure, including systolic blood pressure (SBP) and diastolic blood pressure (DBP), was manually recorded 3–4 times following the requirement of 5 minutes of quiet rest in a seated position and the determination of the maximum inflation level.

### Statistical analysis

According to the complex survey design, all analyses in the current study were weighted by MEC exam weights and employed NHANES strata and population sampling units (40–42). Weighted means (standard error, SE) were utilized for continuous variables, and weighted proportions were utilized for categorical variables. The weighted linear regression model was used to determine the associations between insulin level and three types of physical activity: work activity, recreational activity, and sedentary behavior. For the crude model, no confounding factors were adjusted. For model 1, age, sex, and race/ethnicity were adjusted. For model 2, BMI categories, education, marital status, poverty status<1.3, SBP, DBP, total cholesterol, HDL-c, LDL-c, glucose, triglycerides, smoking status, and alcohol drinking status were additionally adjusted based on model 1. Statistical significance was set at P value < 0.05. All statistical analyses were performed using R (version 4.2.0, http://www.R-project.org, The R Foundation).

## Results

### Baseline participant characteristics

**Figure 1:**
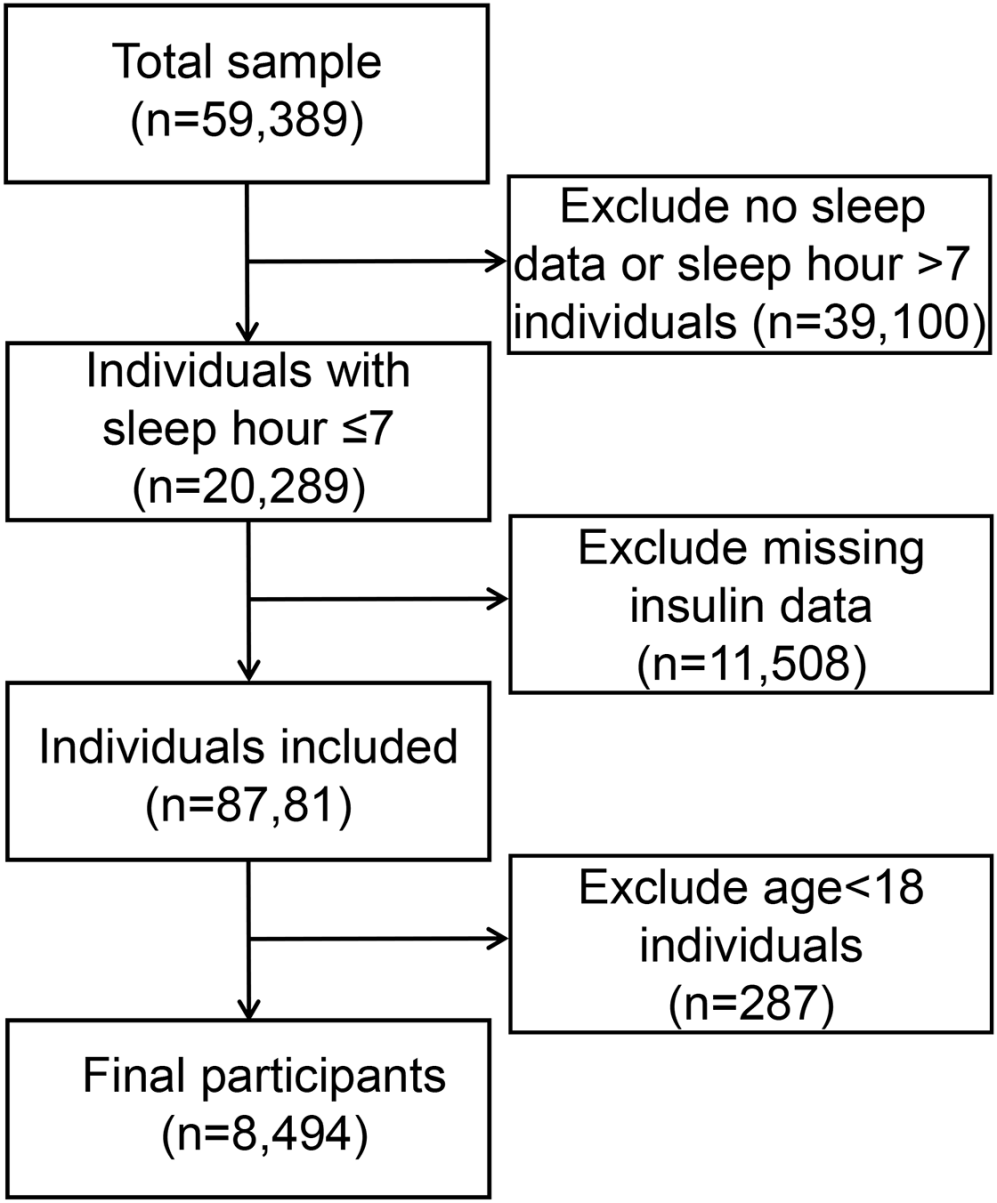
Flow chart of the process of data filtering.

Out of the 59,389 participants (representing a weighted population of 55,989,817) in the 2007-2018 NH ANES cycles, 20,289 were identified as short sleepers (< 7 hours per night). Among these short sleepers, we included 8,494 participants aged ≥18 years with available insulin data. **Figure 1** illustrates the detailed flow chart of the process of data filtering. The baseline clinical and demographic features are presented in **Table 1**, with the participants’ weighted characteristics sub-classified based on sex. The mean values of fasting glucose, insulin, total glucose, blood pressure, and diastolic blood pressure were significantly lower in females than in males (P < 0.001). However, females have a statistically significantly higher mean on HDL-C and TC than males (P < 0.001). Males were younger (P < 0.001) and had a higher proportion of smokers, alcohol consumption, poverty income ratio 3, married status or living with a partner, BMI between 25 and 30, non-Hispanic white and Mexican American (P < 0.001) compared to participants in female group, but a lower proportion of non-Hispanic black, unmarried or divorced status, poverty income ratio< 1 and BMI< 25 or ≥30 (There were no statistically significant differences in education, diabetes, or LDL-c (P > 0.05).

**Table 1:** Weighted characteristics of study populations.

Variable	All participants	Male		Female		*P-value*
Age						<0.001
<44	44.40	45.17		43.57		
[44, 60)	32.71	34.21		31.10		
≥60	22.90	20.62		25.32		
Race/ethnicity						<0.001
Non-hispanic White	65.43	67.26		63.47		
Non-hispanic Black	11.70	10.05		13.47		
Mexican American	8.50	9.12		7.84		
Other Race/ethnicity	14.37	13.57		15.22		
Marital status						<0.001
Never married	17.33	18.21		16.40		
Married/living with partner	63.97	68.35		59.32		
Widowed/divorced	18.70	13.44		24.29		
Poverty Income Ratio						<0.001
<1	15.33	12.87		17.98		
[1,3)	36.05	34.72		37.49		
≥3	48.62	52.41		44.53		
Education						0.056
Below high school	5.43	5.46		5.41		
High school	35.97	37.26		34.60		
College or above	58.59	57.28		59.99		
Smokers						<0.001
Never smoker	54.98	48.24		62.12		
Former smoker	24.19	28.90		19.19		
Current smoker	20.83	22.85		18.69		
Alcohol drinkers						<0.001
Nondrinker	32.88	27.22		38.91		
Moderate alcohol use	49.88	52.69		46.87		
High alcohol use	17.25	20.09		14.22		
BMI						<0.001
<25	29.81	26.95		32.85		
[25, 30)	33.15	38.96		26.96		
≥30	37.04	34.09		40.19		
Diabetes mellitus						0.242
No	80.73	80.07		81.43		
Yes	19.27	19.93		18.57		
FG (mmol.L)	5.93	6.09		5.75		<0.001
Insulin (pmol.L)	81.05	85.03		76.80		<0.001
TG (mg.dl)	125.90 ± 1.61	136.00 ± 2.27		115.14 ± 2.12		<0.001
TC (mg.dl)	191.33 ± 0.59	188.53 ± 0.59		194.32 ± 0.73		<0.001
HDL-c (mg.dl)	53.26 ± 0.28	48.41 ± 0.31		58.43 ± 0.40		<0.001
LDL-c (mg.dl)	113.50 ± 0.47	113.73 ± 0.63		113.26 ± 0.62		0.574
SBP (mmHg)	120.52 ± 0.25	122.26 ± 0.29		118.64 ± 0.36		<0.001
DBP (mmHg)	70.12 ± 0.22	71.79 ± 0.25		68.32 ± 0.29		<0.001

Mean ± SE for continuous variables: P-value was calculated by weighted linear regression model. % for categorical variables: P-value was calculated by weighted chi-square test. BMI, body mass index; FG, fast glucose; TG, triglyceride; TC, total cholesterol; HDL-c, high-density lipoprotein cholesterol; LDL-c, low-density lipoprotein cholesterol; SBP, systolic blood pressure; DBP, diastolic blood pressure.

### The association between PA, SB and the level of insulin in short sleepers

Data from the multivariate regression analysis are shown in **Table 2**. In the crude model, work activity and recreational activity were negatively associated with insulin [β value 95% CI = -0.002 (-0.002, -0.001), β value 95% CI = -0.012 (-0.017, -0.007), respectively], while sedentary behavior was positively correlated with insulin [β value 95% CI = 0.035 (0.022, 0.049)] in the total population. However, in the female work activity group, there seemed not to be a significant negative correlation with insulin (*P* > 0.05). Even after controlling for potential confounding factors, this relationship was found to persist in model 2 [β value 95% CI = -0.002 (-0.003, 0.001), β value 95% CI = -0.008 (-0.012, -0.003), β value 95% CI = 0.022 (0.009, 0.034)].

**Table 2:** Association between physical activity, sedentary behavior and serum insulin level in short sleepers.

	Crude model^a^		Model 1^b^		Model 2^c^	
	OR (95% CI)	*P-value*	OR (95% CI)	*P-value*	OR (95% CI)	*P-value*
Work activity (min/week)	-0.002 (-0.004,-0.001)	0.009	-0.003 (-0.005,-0.001)	0.003	-0.002 (-0.003, 0.001)	0.015
Recreational activity (min/week)	-0.012 (-0.017,-0.007)	<0.001	-0.013 (-0.019,-0.008)	<0.001	-0.008 (-0.012,-0.003)	0.001
Sedentary behavior (min/day)	0.035 (0.022,0.049)	<0.001	0.039 (0.025, 0.052)	<0.001	0.022 (0.009, 0.034)	0.001
Male						
Work activity (min/week)	-0.003 (-0.006,-0.001)	0.006	-0.003 (-0.006,-0.001)	0.005	-0.002 (-0.004, 0.001)	0.057
Recreational activity (min/week)	-0.012 (-0.020,-0.005)	0.002	-0.013 (-0.020,-0.006)	0.001	-0.007 (-0.013,-0.002)	0.014
Sedentary behavior (min/day)	0.044 (0.022,0.066)	<0.001	0.05 (0.027, 0.073)	<0.001	0.032 (0.010, 0.055)	0.006
Female						
Work activity (min/week)	-0.001 (-0.003,0.002)	0.468	-0.001 (-0.004, 0.001)	0.337	-0.001 (-0.003, 0.001)	0.223
Recreational activity (min/week)	-0.014 (-0.021,-0.006)	0.001	-0.014 (-0.022,-0.006)	0.001	-0.008 (-0.015,-0.002)	0.015
Sedentary behavior (min/day)	0.023 (0.012,0.035)	<0.001	0.025 (0.014, 0.036)	<0.001	0.009 (-0.001, 0.019)	0.096

^a^Crude model, no covariates were adjusted. ^b^Model 1, age, sex, race/ethnicity were adjusted. ^c^Model 2, age, sex, race/ethnicity, body mass index, education marital status, poverty status, FG, TG, TC, HDL-c, LDL-c, SBP, DBP, smoking status, and alcohol drinking status were adjusted. OR, odds ratio; CI, confidence interval.

**Figure 2:**
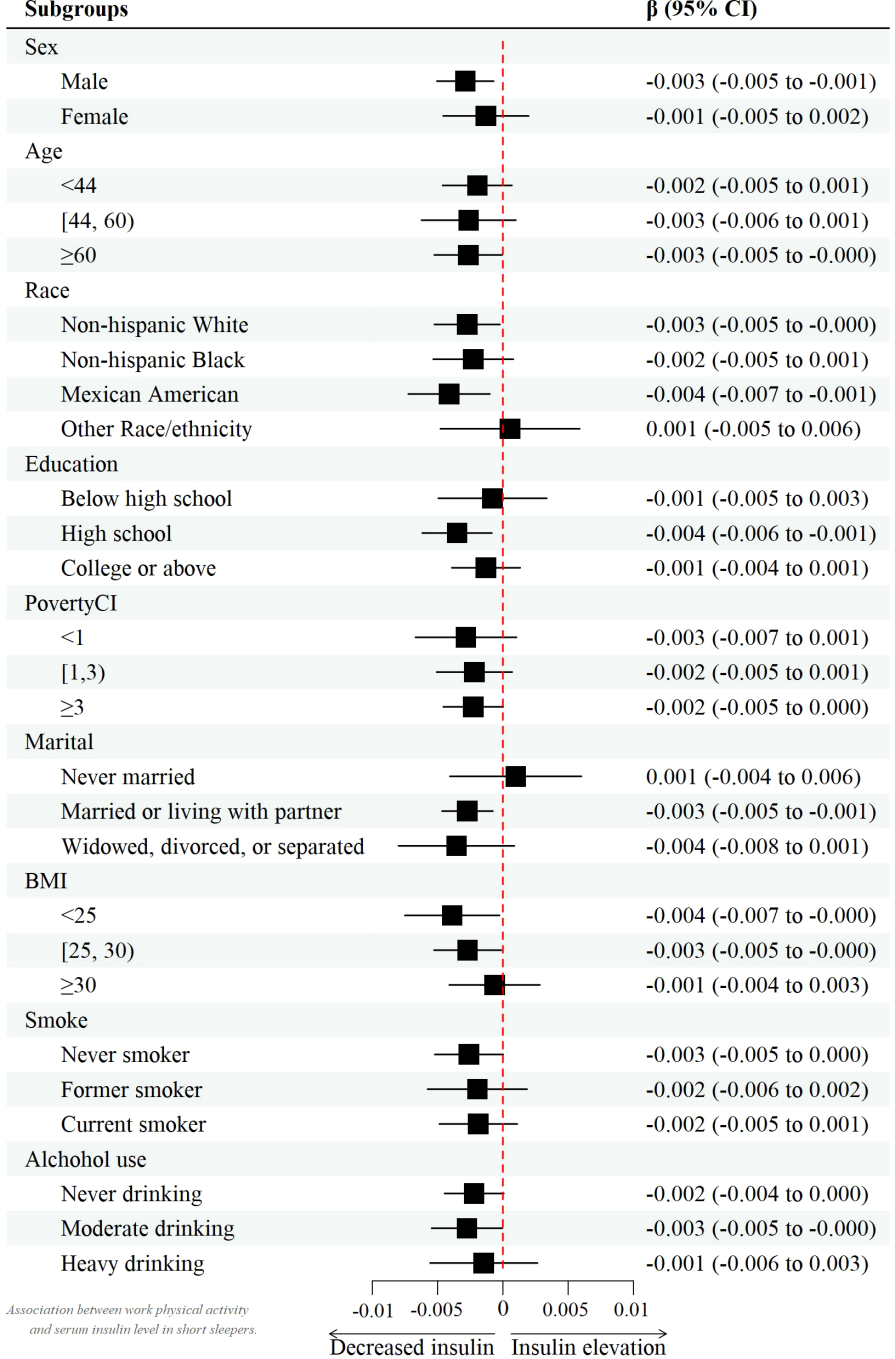
Forest plot of the effect of work activity on insulin.

**Figure 3:**
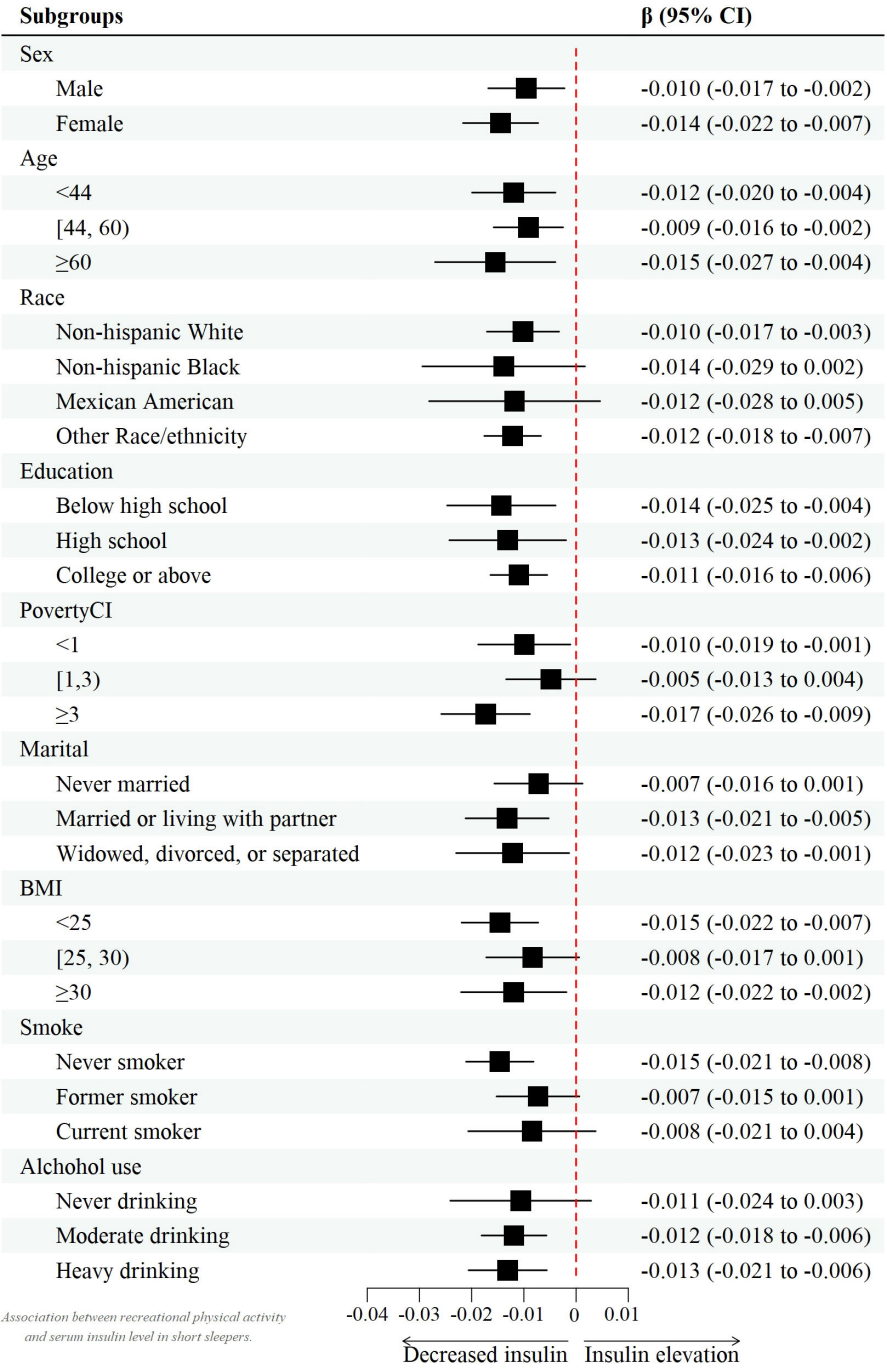
Forest plot of the effect of recreational activity on insulin.

**Figure 4:**
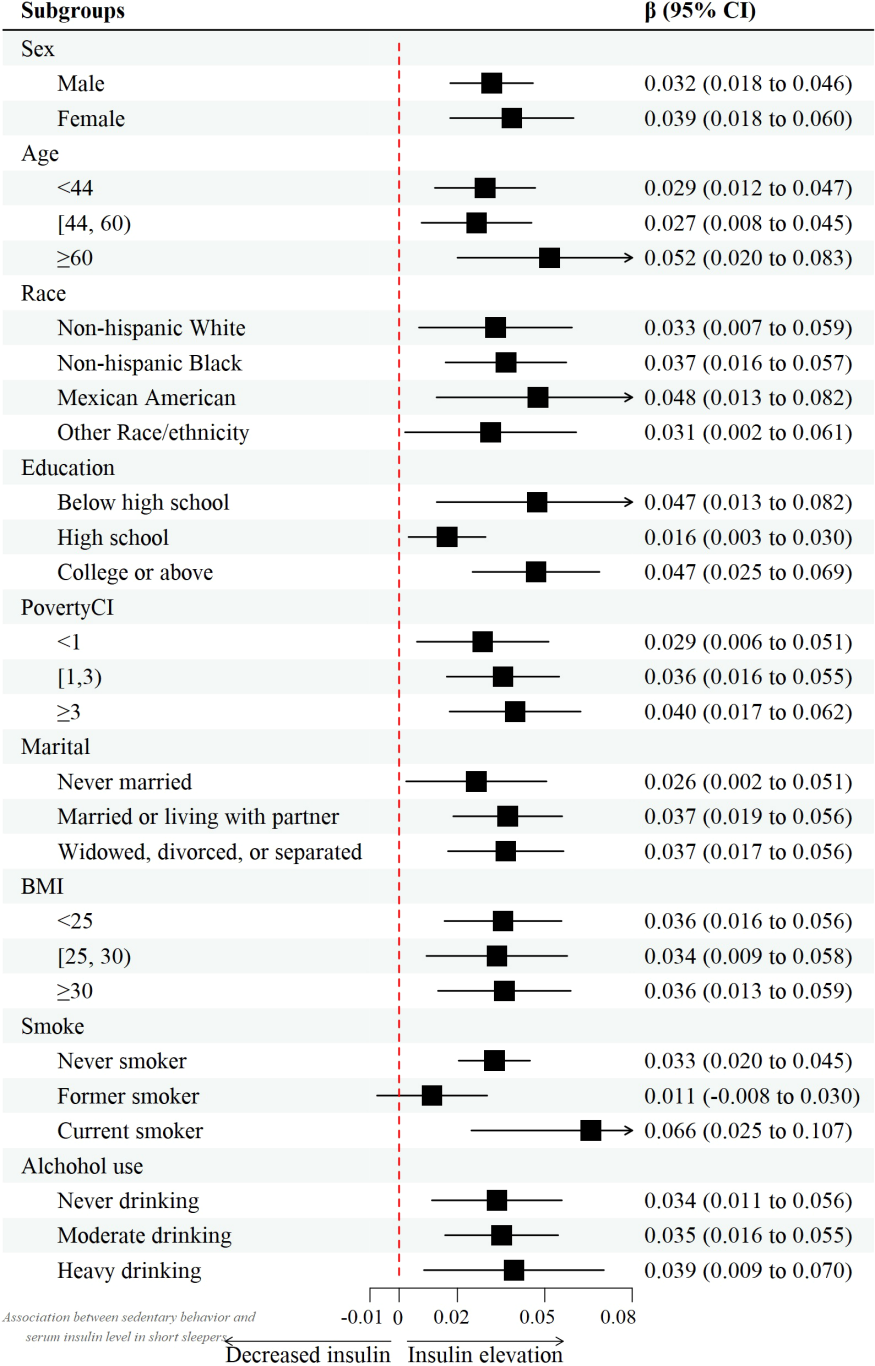
Forest plot of the effect of sedentary behavior on insulin.

However, after controlling for confounders, this association only persisted in male leisure sport and sedentary behavior when grouped by sex. (model 2) [β value 95% CI = -0.007 (-0.013, -0.002), β value 95% CI =0.032 (0.010, 0.055), respectively] and female recreational activity [β value 95% CI =-0.008 (-0.015, -0.002)]. **Figures 2**–**4** show the results of the subgroup analyses on the effect of work activity, recreational activity and sedentary behaviors on insulin levels, respectively. 

### Sensitivity analysis in participants without DM

DM should always be identified as a major covariate. A diabetes-specific awareness evaluation was carried, and the relationship between regular exercise and insulin in short sleepers was observed among participants without have diabetes. As determined by multivariate linear regression, work activity and leisure activity were negatively associated with glycemia in attendees without hyperglycemia of both sexes.[β value 95% CI = -0.001 (−0.003, 0.000), β value 95% CI = -0.005 (-0.009, -0.001), respectively]. In spite of this, in all participants without DM only recreational activity was associated with lower insulin levels in those without diabetes mellitus after controlling for confounding variables.[Model 2, β value 95% CI =-0.005 (-0.008, -0.002)]. Males were also found to have this association when grouped by sex. [β value 95% CI =-0.006 (-0.010, -0.002)]. Moreover, sedentary behavior was positively correlated with insulin levels in this group [β value 95% CI =0.011 (0.001,0.021)]. However, according to the analytical results in females, there was no significant positive or negative association among all three groups (*P* > 0.05). **Table 3** demonstrates the association between work activity, recreational activity, sedentary behavior, and serum insulin levels in short sleepers without DM.

**Table 3:** Association between physical activity, sedentary behavior and serum insulin level in short sleepers without DM.

	Crude model^a^		Model 1^b^		Model 2^c^	
	OR (95% CI)	*P-value*	OR (95% CI)	*P-value*	OR (95% CI)	*P-value*
Work activity (min/week)	-0.001 (-0.003,0.000)	0.042	-0.001 (-0.002, 0.001)	0.414	-0.001 (-0.002, 0.001)	0.414
Recreational activity (min/week)	-0.005 (-0.009,-0.001)	0.026	-0.007 (-0.011,-0.003)	0.002	-0.005 (-0.008,-0.002)	0.003
Sedentary behavior (min/day)	0.008 (-0.001, 0.018)	0.079	0.011 (0.002, 0.020)	0.017	0.006 (-0.002, 0.013)	0.176
Male						
Work activity (min/week)	-0.002 (-0.004,-0.000)	0.017	-0.003 (-0.004,-0.001)	0.005	0.000 (-0.002, 0.002)	0.852
Recreational activity (min/week)	-0.006 (-0.011,-0.001)	0.026	-0.008 (-0.013,-0.002)	0.008	-0.006 (-0.010, -0.002)	0.004
Sedentary behavior (min/day)	0.005 (-0.007, 0.018)	0.400	0.008 (-0.004, 0.021)	0.210	0.011 (0.001, 0.021)	0.038
Female						
Work activity (min/week)	0.000 (-0.002, 0.002)	0.697	-0.001 (-0.003,0.001)	0.410	-0.001 (-0.002, 0.000)	0.128
Recreational activity (min/week)	-0.004 (-0.009, 0.001)	0.154	-0.006 (-0.011,-0.001)	0.032	-0.004 (-0.008, 0.001)	0.126
Sedentary behavior (min/day)	0.011 (-0.002, 0.024)	0.110	0.015 (0.001, 0.028)	0.038	0.000 (-0.010, 0.010)	0.947

^a^Crude model, no covariates were adjusted. ^b^Model 1, age, sex, race/ethnicity were adjusted. ^c^Model 2, age, sex, race/ethnicity, body mass index, education marital status, poverty status, FG, TG, TC, HDL-c, LDL-c, SBP, DBP, smoking status, and alcohol drinking status were adjusted. OR, odds ratio; CI, confidence interval.

### The Effect of PA, SB and lipid indices (LDL-C, HDL-C, FG, TC and TG) interaction on insulin levels in short sleepers

Shows in **Table 4** that different quantile levels of physical activity and lipid indices (LDL-c, TC, TG and HDL-c) have different effects on insulin levels. First, only the recreational activity group showed significant differences in the lower HDL-c quantile, with β values. 95% CI =−0.010 (−0.017, −0.003) in the adjusted multivariate regression model. In addition to recreational activity, Insulin levels in the HDL-c tertile 2 were found to be linked to inactivity, according with study. [(β-value. 95% CI =0.019 (0.005,0.032)].

**Table 4:** The effect of PA and lipid indices (LDL-c, HDL-c, FG, TC and TG) interaction on the insulin levels in short sleepers.

Physical activity	Tertile 1 of HDL-c		Tertile 2 of HDL-c		Tertile 3 of HDL-c	
	β (95% CI)	*P-value*	β (95% CI)	*P-value*	β (95% CI)	*P-value*
Work activity (min/week)	-0.002 (-0.006, 0.001)	0.151	-0.001 (-0.003, 0.001)	0.163	-0.001 (-0.002, 0.000)	0.147
Recreational activity (min/week)	-0.010 (-0.017,-0.003)	0.006	-0.010 (-0.016,-0.004)	0.002	-0.004 (-0.011, 0.003)	0.241
Sedentary behavior (min/day)	0.035 (0.000, 0.069)	0.052	0.019 (0.005, 0.032)	0.008	0.010 (0.000, 0.020)	0.046
Physical activity	Quantile 1 of LDL-c		Quantile 2 of LDL-c		Quantile 3 of LDL-c	
	β (95% CI)	*P-value*	β (95% CI)	*P-value*	β (95% CI)	*P-value*
Work activity (min/week)	-0.001 (-0.003, 0.001)	0.424	-0.002 (-0.004, 0.000)	0.033	-0.003 (-0.006, 0.001)	0.146
Recreational activity (min/week)	0.003 (-0.005, 0.010)	0.490	-0.015 (-0.022,-0.008)	<0.001	-0.014 (-0.022,-0.006)	0.002
Sedentary behavior (min/day)	0.027 (-0.007, 0.062)	0.122	0.014 (-0.004, 0.032)	0.121	0.022 (0.009, 0.036)	0.002
Physical activity	Quantile 1 of TC		Quantile 2 of TC		Quantile 3 of TC	
	β (95% CI)	*P-value*	β (95% CI)	*P-value*	β (95% CI)	*P-value*
Work activity (min/week)	-0.002 (-0.004, 0.000)	0.033	-0.002 (-0.005, 0.001)	0.129	-0.001 (-0.005, 0.002)	0.385
Recreational activity (min/week)	0.001 (-0.006, 0.007)	0.929	-0.01 (-0.015,-0.005)	<0.001	-0.015 (-0.024,-0.006)	0.001
Sedentary behavior (min/day)	0.014 (-0.013, 0.041)	0.329	0.028 (0.004, 0.052)	0.026	0.018 (0.007, 0.028)	0.002
Physical activity	Quantile 1 of TG		Quantile 1 of TG		Quantile 1 of TG	
	β (95% CI)	*P-value*	β (95% CI)	*P-value*	β (95% CI)	*P-value*
Work activity (min/week)	-0.002 (-0.003, 0.000)	0.028	-0.002 (-0.004,-0.001)	0.007	-0.002 (-0.006, 0.002)	0.283
Recreational activity (min/week)	-0.002 (-0.007, 0.003)	0.445	-0.006 (-0.011, 0.000)	0.061	-0.018 (-0.027,-0.009)	<0.001
Sedentary behavior (min/day)	0.005 (-0.008, 0.019)	0.436	0.019 (0.006, 0.033)	0.007	0.036 (0.002, 0.069)	0.041
Physical activity	Quantile 1 of FG		Quantile 1 of FG		Quantile 1 of FG	
	β (95% CI)	*P-value*	β (95% CI)	*P-value*	β (95% CI)	*P-value*
Work activity (min/week)	0.000 (-0.002, 0.001)	0.598	-0.001 (-0.003, 0.000)	0.040	-0.003 (-0.008, 0.001)	0.147
Recreational activity (min/week)	-0.004 (-0.008, 0.000)	0.069	-0.007 (-0.011,-0.002)	0.005	-0.015 (-0.033, 0.003)	0.112
Sedentary behavior (min/day)	0.009 (-0.001, 0.019)	0.079	0.006 (-0.004, 0.016)	0.252	0.054 (0.019, 0.089)	0.004

The model was adjusted for age, sex, race/ethnicity, body mass index, education marital status, poverty status, SBP, DBP, total cholesterol, HDL-c, LDL-c, glucose, triglycerides, smoking status, alcohol drinking status, HDL-c, LDL-c, TC, TG, and FG. In the subgroup analysis stratified by HDL-c, LDL-c, TC, TG, and FG quantile, the model was not adjusted for HDL-c, LDL-c, TC, TG, and FG, respectively.

However, in the highest HDL-c tertile, only sedentary behavior was positively correlated with insulin levels [β value 95% CI =0.010 (0.000,0.020)]. Second, no significant positive or negative association was observed among all three groups (*P* > 0.05) with insulin levels in the lower LDL-c quantile. However, a significant negative association was observed for work activity [β value 95% CI =-0.002 (-0.004, 0.000)] and recreational activity [β value 95% CI =-0.015 (-0.022, -0.008)]. Similarly, we found a significant negative association between recreational activity and insulin levels [β value 95% CI =-0.014 (-0.022, -0.006)] and a positive association with sedentary behavior [β value 95% CI =0.022 (0.009, 0.036)]. Third, lower TC and TG tertile presented the same trend. Namely, only work activity was negatively correlated with insulin levels [β value 95% CI =−0.002 (−0.004,0.000), β value 95% CI =-0.002 (-0.003,0.000), respectively]. Whereas sedentary behavior was positively correlated with insulin levels [β value 95% CI =0.028 (0.004,0.052), β value 95% CI =0.019 (0.006,0.033), respectively] in this group, work activity was significantly negatively correlated with insulin levels in TG [β value 95% CI =-0.002 (-0.004, -0.001)] while TC was not. In contrast, recreational activity was significantly negatively correlated with insulin levels in TC [β value 95% CI =-0.01 (-0.015, -0.005)], while TG was not. Simultaneously, upper TC and TG quantiles also presented the same trend, namely, recreational activity was significantly negatively correlated with insulin levels [β value 95% CI =−0.015 (−0.024, -0.006), β value 95% CI =-0.018 (-0.027, -0.009), respectively], and a positive association [β value 95% CI =0.018 (0.007, 0.028), β value 95% CI = 0.036 (0.002, 0.069), respectively] was observed in sedentary behavior. Last but not least, similar to the lower LDL-c quantile, all three groups had no significant positive or negative association (*P* > 0.05) with insulin levels in the lower FG quantile. Nevertheless, work activity and recreational activity presented a significant negative association with insulin level [β value 95% CI = -0.001 (-0.003,0.000), β value 95% CI =-0.007 (-0.011, -0.002), respectively]. In addition, only sedentary behavior had significant differences in the sedentary behavior group, with β values 95% CI = 0.054 (0.019,0.089).

## Discussion

To our knowledge, this is the first study to show the association between different forms of PA, SB and insulin levels in short sleepers, and we further analyzed this association based on characteristics of the large included population, conducted a sensitivity analysis in participants’ diabetes mellitus, and explored the effect of PA and different levels of lipid indices (LDL-C, HDL-C, FG, TC and TG). A previous study confirmed that PA can improve IR (43). Herein, we could regard sedentary behavior as an extensively low level of PA (44). Because of the findings in this study, our results showed that work and recreational activity were negatively correlated with insulin, while sedentary behavior was the opposite, and this tendency persisted in further stratified analysis. Consequently, even in short sleepers, it’s possible that higher PA densities might dramatically decrease insulin levels. However, the results also showed that recreational activity significantly reduced insulin levels more than work activity. Other studies have also confirmed this view. Namely, it is not enough to rely only on work-related physical activity to maintain fitness, and they have to increase recreational activity to ensure the effect of preventing a variety of chronic diseases (45, 46).

Although plentiful evidence showed that SB exerted great harm to the body, increasing the risk of multiple diseases and mortality (47, 48), the estimated prevalence of prolonged sitting per day generally remained high and stable, approximately 62% [95% CI, 58% to 66%] in US adults (49). Provided that plus the hazard risk caused by insufficient sleep, there is no doubt that the risk of multiple diseases will increase (50, 51). Our results also reflected this fact, while it was interesting that female SB seemed not to be significantly positively correlated with insulin levels in the adjusted model, which coincided with previous research results (52). Insulin sensitivity and SB may be influenced by estrogen and progesterone, which have the possibilities to govern neuro transmitters and maintain beta-cell competence, which could account for this finding (53, 54). This conclusion also applied to participants without diabetes mellitus in the sensitivity analysis after adjusting for confounding factors. Thus, it could be seen that males without diabetes should further reduce sedentary behavior to prevent the occurrence of IR.

In contrast, both recreational and work activities were significantly positively correlated with insulin levels in all participants. Unlike sedentary behavior, recreational activity was significantly associated with insulin levels in both males and females. However, work activity did not seem to have such a significant trend in all genders, even in this particular but enormous population that possessed insufficient sleep. Generally, leisure time is a major part of PA and the amount of activity generated by work is insufficient to maintain basic PA needs (45, 55, 56). Additionally, among females without diabetes, their recreational activity level was insignificantly correlated with insulin level. In addition to the reasons mentioned above, there are sex differences in substrate utilization during PA (57). As a result, more research is needed to determine the role of sexual hormones or sex discrepancies inside this route.

Among the explorations of the effect of PA and several lipid index interactions on insulin levels in short sleepers, we found that low or middle levels of HDL-C seemed to have a significant association with PA, while high levels demonstrated the opposite trend. LDL-C appeared to be the contrary result, with a high level positively correlated with SB A low level negatively correlated with recreational activity. Simultaneously, according to our results, it seemed that when TG was at the medium level, the relationship between PA or SB within lower degrees, and insulin was more significant. In contrast, with the increase in TG level, the greater the intensity of PA, the closer the negative relationship between PA and insulin was; obviously, TC presented similar results. The latest research has shown that insulin resistance affects the occurrence and development of dyslipidemia through several mechanisms, including TG, HDL-c, and LDL-c (58, 59).

Increased HTGL exercise is seen to be heavily associated to IR, which could lead to a faster HDL-C clearance and a reduction in HDL-C levels (52, 60). In addition, we can observe that when fast blood glucose (FG) is at a medium-high level, the intensity of PA shows a significant negative correlation with it. At the same time, SB would further significantly increase the body content of FG, which does not also increase the possibility of occurrence of IR but also a powerful independent risk marker for cardiovascular diseases (CVD) and contributes to the increased atherosclerotic risk in diabetes mellitus (DM) (61, 62). The latest study claimed that a high binding affinity was observed in patients with T2DM samples concerning healthy subjects against Methylglyoxal glycated fibrinogen antigen compared to native fibrinogen, which might explain this phenomenon to some extent (62). The above mentioned insulin resistance and dyslipidemia are all risk factors for CVD and DM. Based on our findings, the intensity and duration of PA can prevent these phenomena because the significant negative association between LDL-c, TG and PA is observed. This is consistent with recent research results, namely, moderate-intensity aerobic exercise can significantly improve insulin sensitivity, Each time about more than 30 minutes, three times a week for a minimum of eight weeks (54, 63).

Consequently, many chronic diseases have been linked to a lack of sleep, and we found that even short sleepers can benefit from the therapeutic benefits of PA, this conclusion is still suitable. In many cases, people lack a comfortable environment that can provide enough sleep duration and good sleep quality (64), or those short sleepers due to various reasons, such as anxiety and depression, can not only mitigate the severity of these emotional diseases (65) but also prevent the occurrence of CVD and DM by conducting regular moderate-intensity aerobic exercise. Additionally, PA usually has no other adverse effects and can cure multiple health concerns immediately, which is the most cost-effective means of maintaining health (66).

This study had several limitations that must be acknowledged. First, since the statistical analysis was based solely on cross-sectional data, it is challenging to establish causality between physical activity (PA) and insulin levels. To strengthen these findings, larger population-based, high-quality randomized controlled trials or longitudinal cohort studies are needed to confirm these conclusions (67). Second, several potential confounding factors were not fully accounted for, such as underlying disorders, dietary habits, and medications, particularly hypoglycemic agents. In NHANES, dietary habits are assessed only once at baseline, without information on changes over time, which limits the accuracy of dietary data. Furthermore, the use of self-reported dietary information introduces recall bias. Third, most measurements of work activity, sedentary behavior, and recreational activity were based on self-reports, which may not be entirely accurate. Future research should consider incorporating more objective methods, such as accelerometers, to capture the volume and intensity of PA with greater precision.

## Conclusions

This study explored the association between physical activity, sedentary behavior and insulin levels in short sleepers for the first time and demonstrated that work activity and recreational activity were negatively correlated with insulin. In contrast, sedentary behavior was the opposite in this population. The sensitivity analysis further confirmed that males without diabetes should especially focus on reducing sedentary behavior to prevent insulin resistance. Short sleepers with regular moderate-intensity aerial exercise can not only mitigate the severity but also prevent the occurrence of CVD and DM, making it an effective approach to maintaining health. Based on our results, further large-scale, high-quality randomized controlled trials are needed to be carried out to confirm the conclusion and further explore the association in the future.

## Data Availability

The datasets presented in this study can be found in online repositories. The names of the repository/repositories and accession number(s) can be found in the article/supplementary material.
